# Prognostic factors in stage I gastric cancer: A retrospective analysis

**DOI:** 10.1515/med-2020-0164

**Published:** 2020-08-03

**Authors:** Dingcheng Zheng, Bangsheng Chen, Zefeng Shen, Lihu Gu, Xianfa Wang, Xueqiang Ma, Ping Chen, Feiyan Mao, Zhiyan Wang

**Affiliations:** Department of General Surgery, HwaMei Hospital, University of Chinese Academy of Sciences, Ningbo, Zhejiang, China; Key Laboratory of Diagnosis and Treatment of Digestive System Tumors of Zhejiang Province, Ningbo, Zhejiang, China; Ningbo Clinical Research Center for Digestive System Tumors, Ningbo, Zhejiang, China; Emergency Medical Center, Ningbo Yinzhou No. 2 Hospital, Ningbo, Zhejiang, China; Department of General Surgery, Zhejiang University School of Medicine Sir Run Run Shaw Hospital, Hangzhou, Zhejiang, China; Department of General Surgery, Zhuji People’s Hospital, Shaoxing, Zhejiang, China; Department of General Surgery, Ningbo Yinzhou No. 2 Hospital, 998 North Qianhe Road, Ningbo, Yinzhou District, Zhejiang, China

**Keywords:** Gastric cancer, T1N1, T2N0, risk factor, prognosis

## Abstract

**Purpose:**

The purpose of this research is to investigate the prognostic factors of patients with stage I gastric cancer (GC) and to determine whether adjuvant chemotherapy improves the prognosis for high-risk patients.

**Methods:**

We performed a retrospective analysis at Sir Run Run Shaw Hospital, Zhejiang University School of Medicine, and HwaMei Hospital, University of Chinese Academy of Sciences from January 2001 to December 2015. Cox regression and Kaplan-Meier were used to evaluate the relationship between the patients’ clinicopathologic characteristics and prognosis.

**Results:**

A total of 1,550 patients were eligible for the study. The 5-year disease-free survival (DFS) rate of all enrolled patients was 96.5%. The pT and pN stages were significantly associated with the prognosis. The 5-year DFS rates of the three subgroups (T1N0, T2N0, and T1N1) were 97.8%, 95.7%, and 90.5%, respectively (*p* < 0.001). In the T1N1 subgroup, patients not undergoing chemotherapy showed a lower 5-year DFS rate compared to those undergoing chemotherapy, although the difference was not statistically significant.

**Conclusions:**

Both the pT and pN stages were closely associated with the prognosis of patients with stage I GC. We also found that the danger coefficient of the pN stage was higher than that of the pT stage, and that postoperative adjuvant chemotherapy might be a reasonable approach to improve outcomes of high-risk patients, particularly in the T1N1 group.

## 1 Introduction

In recent decades, the early detection rate of gastric cancers (GCs) has been increasing with the prevalence of endoscopic techniques [1]. According to the eighth edition of the American Joint Committee on Cancer (AJCC) tumor-lymph node-metastasis (TNM) classification, stage I GC has two subtypes: stage IA (invading the mucosa and submucosa, no positive lymph nodes, and no distant metastasis, T1N0M0) and stage IB. The stage IB GC consists of T1N1M0 (invading the mucosa and submucosa, having one to two positive lymph nodes but no distant metastasis) and T2N0M0 (invading the muscularis propria, no positive lymph nodes, and no distant metastasis) [2,3]. Patients with stage I GC typically have an excellent prognosis but there is still a small risk of relapses or distant metastases. Previous studies have also suggested that the prognosis of stage IB patients is worse than that of stage IA patients [4]. Additionally, some scholars believe that the prognosis of patients between T1N1 and T2N0 was different after investigating the prognostic factors of early GC [5,6,7]. Almost high-quality clinical trials of postoperative chemotherapy for GC excluded stage I patients [8,9].

The guidelines can also differ depending on the county; according to the Japanese Gastric Cancer Association treatment guidelines, observation alone is recommended without chemotherapy for stage I GC patients who had undergone curative resection [10], but the National Comprehensive Cancer Network (NCCN) recommends adjuvant chemotherapy for high-risk stage I patients (lymph nodes metastasis, poorly differentiated, lymphovascular invasion, perineural invasion, or under 50 years of age) after curative resection [2]. Although some retrospective small-sample/single-center studies have reported on the risk factors influencing the prognosis in patients with early GC, no general consensus on the management of early GC currently exists [4,11,12]. Furthermore, the definition of stage I GC continues to change with each update of the TNM staging system. Therefore, this study was conducted to investigate the risk factors influencing the prognosis of patients with stage I GC and to evaluate the efficacy of adjuvant chemotherapy for high-risk patients.

## 2 Materials and methods

### 2.1 Study population

**Figure 1:**
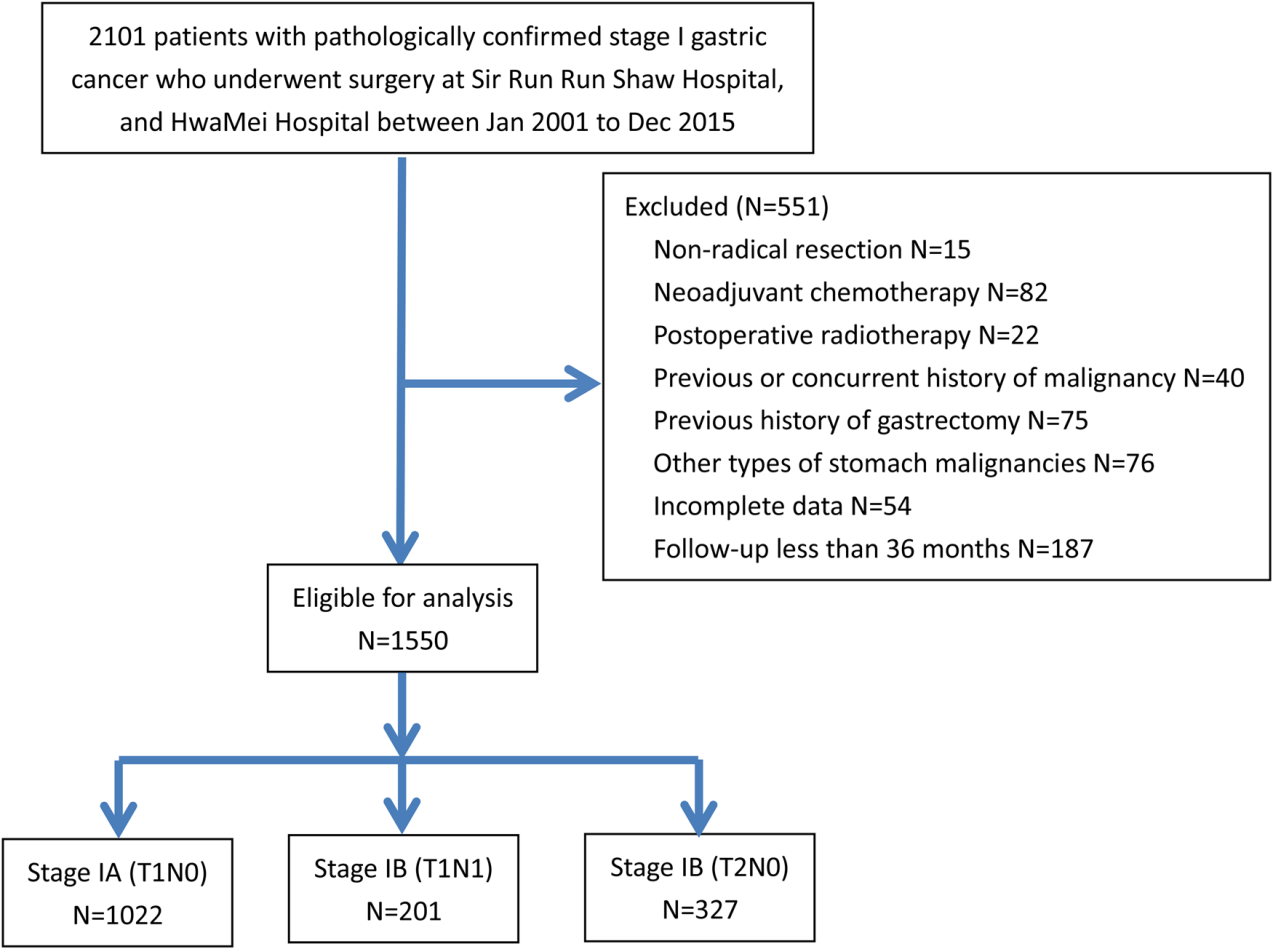
Flowchart of the study population.

All patients diagnosed with stage I GC after surgical resection between January 2001 and December 2015 at Sir Run Run Shaw Hospital, Zhejiang University School of Medicine, and HwaMei Hospital, University of Chinese Academy of Sciences, were extracted from a database specially created for this purpose, and retrospectively analyzed in this study. Figure 1 summarizes the inclusion and exclusion criteria used in this study. Adjuvant chemotherapy was based on 5-fluorouracil (5-FU) or platinum. The surgical specimens were examined by pathologists using the updated edition of the UICC/AJCC TNM staging system, which was then converted to the eighth edition at the time of our analysis. Although no approval number exists due to the particularity of this retrospective research, the ethics committees of both hospitals (Sir Run Run Shaw Hospital, Zhejiang University School of Medicine, and HwaMei Hospital, University of Chinese Academy of Sciences) have approved the implementation of this study. A written consent was obtained from all patients before enrollment.

## 3 Follow-up

The patients were followed up at every 6 months for the first 2 years, and annually thereafter until death or at least 5 years after undergoing curative surgery. Disease-free survival (DFS) was defined as the time from surgery to locoregional recurrence, distant recurrence, or death. Patients for whom none of these events were recorded were censored at the date of their last known contact. The median follow-up time for the entire cohort was 67 months (range 10–209 months) and follow-up of all patients included in this study was concluded in January 2019.

## 4 Statistical analysis

Continuous variables were compared using the independent samples *t-*test or the Wilcoxon rank-sum test, and categorical variables were compared using the Pearson’s chi-squared test or the Fisher’s exact test when appropriate. The potentially relevant factors obtained from the univariate analysis were then assessed in the multivariate model using Cox’s regression. A univariate logistic regression analysis was also performed to evaluate the relationship between the clinicopathological factors and lymph node metastasis (N1)/muscularis propria invasion (T2). The independent risk factors were included in the multivariate logistic regression analysis. Hazard ratios (HRs) and 95% confidence intervals (CIs) were calculated. The DFS rate was calculated using the Kaplan-Meier method, and the log-rank test was employed to determine the significance. All statistical tests were performed two-sided, and a *p* < 0.05 difference was considered statistically significant. Analyses were performed using the SPSS software (version 25.0, SPSS Inc. IL, USA).

## 5 Results

### 5.1 Clinicopathologic characteristics

Between January 2001 and December 2015, there were a total of 2,101 naive patients with stage I GC after surgical resection at Sir Run Run Shaw Hospital, Zhejiang University School of Medicine, and HwaMei Hospital, University of Chinese Academy of Sciences. Of these, 551 patients were excluded from the analysis for previously described reasons (for details, see Figure 1) and 1,550 patients were eligible for analysis in the present study. The 5-year DFS rate of all patients recruited was 96.5%, and 64 patients died due to their GC during the follow-up. The clinicopathologic characteristics of these patients are summarized in Table 1.

**Table 1:** Baseline clinicopathological characteristics of patients with stage I GC

	*N* = 1550
Age (years) (mean ± SD)	58.8 ± 11.0
Gender	
Male	1,221 (78.8%)
Female	329 (21.2%)
Body mass index (kg/m^2^) (mean ± SD)	21.8 ± 2.1
American Society of Anesthesiologists	
1–2	1,121 (72.3%)
3–4	429 (27.7%)
Tumor location	
Upper third	189 (12.2%)
Middle third	129 (8.3%)
Lower third	1,219 (78.6%)
Two thirds or more	13 (0.8%)
Type of gastrectomy	
Distal subtotal	1,245 (80.3%)
Total	257 (16.6%)
Proximal subtotal	48 (3.1%)
Tumor size (cm) (mean ± SD)	3.0 ± 1.5
Histologic type	
Differentiated	912 (58.8%)
Undifferentiated	638 (41.2%)
Perineural invasion	
Absence	1510 (97.4%)
Presence	40 (2.6%)
Lymphovascular invasion	
Absence	1457 (94.0%)
Presence	93 (6.0%)
pT category	
T1	1223 (78.9%)
T2	327 (21.1%)
pN category	
N0	1349 (87.0%)
N1	201 (13.0%)
Chemotherapy	
No	1118 (72.1%)
Yes	432 (27.9%)
Number of the examined lymph nodes (mean ± SD)	26.6 ± 13.0

### 5.2 Prognostic factors and survival analysis

**Figure 2:**
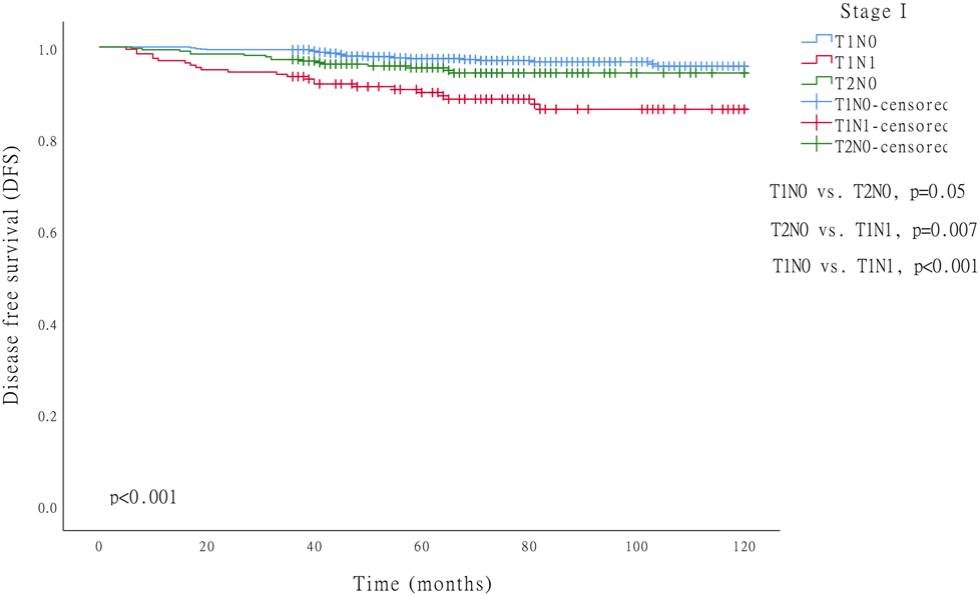
Comparison of survival curves in patients with stage I GC (*p* < 0.001).

The multivariate Cox proportional hazards model analysis showed that both the pT stage and the pN stage were the independent prognostic factors (Table 2). As both pT and pN stages were significantly associated with the prognosis, according to the TNM staging system (eighth edition), all patients were divided into three subgroups: T1N0 (stage IA), T1N1 (stage IB), and T2N0 (stage IB). The 5-year DFS rate of 1,022 patients with T1N0 was 97.8%, and the median follow-up duration was 66 months (range 22–209). The 5-year DFS rate for the 201 T1N1 patients was 90.5%, while the median follow-up duration was 75 months (range 10–207). As for the 327 T2N0 patients, the 5-year DFS rate was 95.7%, and the median follow-up duration was 66 months (range 23–208) (*p* < 0.001) (Figure 2).

**Table 2:** Univariate and multivariate analyses of 5-year DFS rate for patients with stage I GC

Clinicopathological feature	Univariate analysis			Multivariate analysis		
	HR	95% CI	*p* value	HR	95% CI	*p* value
Age (years)						
≤ 60	1					
> 60	0.63	0.37–1.05	0.075			
Gender						
Male	1					
Female	0.96	0.53–1.72	0.880			
Body mass index (kg/m^2^)						
<24	1					
≥24	0.99	0.86–1.34	0.771			
American Society of Anesthesiologists						
1–2	1					
3–4	1.51	0.78–2.45	0.256			
Tumor location						
Upper third						
Middle third						
Lower third						
Two thirds or more						
Type of gastrectomy						
Distal subtotal	1					
Total	0.32	0.12–0.88	0.028			
Proximal subtotal	1.85	0.67–5.10	0.233			
Tumor size						
≤3.0 cm	1					
>3.0 cm	0.49	0.12–1.98	0.313			
Histologic type						
Differentiated	1					
Undifferentiated	1.13	0.70–1.83	0.608			
Perineural invasion						
Absence	1					
Presence	1.30	0.32–5.31	0.717			
Lymphovascular invasion						
Absence	1					
Presence	1.07	0.39–2.94	0.897			
pT category						
T1	1			1		
T2	1.34	0.78–2.29	0.291	2.42	1.32–4.44	0.004
pN category						
N0	1			1		
N1	3.33	2.01–5.50	<0.001	4.23	2.42–7.39	<0.001
Chemotherapy						
No	1					
Yes	1.98	1.23–3.20	0.005			
Number of the examined lymph nodes						
≤15	1					
>15	1.03	0.57–1.85	0.935			

### 5.3 Subgroup analysis for stage IB

**Figure 3:**
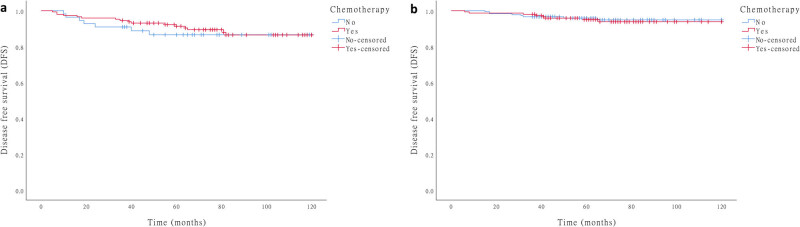
Prognostic impact of adjuvant chemotherapy in patients with stage IB GC, (a) T1N1, *p* = 0.641; (b) T2N0, *p* = 0.781.

The 5-year DFS rate between patients with T1N1 and T2N0 was statistically significant (*p* = 0.018). Additionally, some clinicopathologic features differed between the two groups, including age, gender, tumor location, size, perineural invasion, and chemotherapy (Supplementary Table S1). Furthermore, the effect of chemotherapy on the prognosis of stage IB patients was analyzed, and it was found that patients with T1N1 GC who did not undergo chemotherapy had a lower 5-year DFS rate compared with T1N1 patients who underwent chemotherapy, but the difference had no statistical significance. It was also found that chemotherapy had no effect on the prognosis in T2N0 patients (Figure 3).

### 5.4 Relationship between lymph node metastasis (N1)/muscularis propria invasion (T2) and clinicopathological characteristics

The multivariate analysis revealed that lymph node metastasis was associated with younger age, female, larger tumor, and lymphovascular invasion (Table 3), and GC with muscularis propria invasion was closely related to older age, larger tumor, non-lower third tumor, and perineural invasion (Table 4).

**Table 3:** Logistic analysis of clinicopathological features associated with lymph node metastasis (pN1)

	Univariate analysis			Multivariate analysis		
	OR	95% CI	*p* value	OR	95% CI	*p* value
Age (years)						
≤60	1			1		
>60	0.66	0.49–0.90	0.008	0.663	0.48–0.91	0.011
Gender						
Male	1			1		
Female	1.66	1.19–2.31	0.003	1.59	1.14–2.23	0.007
Body mass index (kg/m^2^)						
< 24	1					
≥ 24	1.29	0.76–2.04	0.915			
American Society of Anesthesiologists						
1–2	1					
3–4	1.21	0.48–2.45	0.621			
Tumor size						
≤3.0 cm	1			1		
>3.0 cm	1.85	1.08–3.19	0.026	1.92	1.10–3.35	0.023
Tumor location						
Upper third	1					
Middle third	1.53	0.72–3.24	0.271			
Lower third	1.87	1.08–3.24	0.027			
Two thirds or more	2.11	0.43–10.41	0.360			
Histologic type						
Differentiated	1					
Undifferentiated	1.27	0.94–1.71	0.115			
Lymphovascular invasion						
Absence	1			1		
Presence	2.52	1.54–4.11	<0.001	2.41	1.47–3.97	0.001
Perineural invasion						
Absence	1					
Presence	NA					

**Table 4:** Logistic analysis of clinicopathological features associated with muscularis propria invasion (pT2)

	Univariate analysis			Multivariate analysis		
	OR	95% CI	*p* value	OR	95% CI	*p* value
Age (years)						
≤60	1			1		
>60	1.72	1.35–2.20	<0.001	1.74	1.34–2.26	<0.001
Gender						
Male	1			1		
Female	0.86	0.63–1.17	0.330			
Body mass index (kg/m^2^)						
<24	1					
≥24	0.93	0.81–1.04	0.829			
American Society of Anesthesiologists						
1–2	1					
3–4	0.88	0.56–1.45	0.683			
Tumor size						
≤3.0 cm	1			1		
>3.0 cm	3.06	1.96–4.77	<0.001	2.92	1.78–4.79	<0.001
Tumor location						
Upper third	1			1		
Middle third	1.04	0.64–1.68	0.879	1.11	0.68–1.83	0.677
Lower third	0.48	0.34–0.67	<0.001	0.51	0.36–0.73	<0.001
Two thirds or more	0.96	0.28–3.23	0.942	0.42	0.11–1.57	0.198
Histologic type						
Differentiated	1					
Undifferentiated	0.86	0.67–1.10	0.225			
Lymphovascular invasion						
Absence	1					
Presence	1.33	0.82–2.14	0.252			
Perineural invasion						
Absence	1			1		
Presence	9.45	4.75–18.80	<0.001	11.60	5.72–23.52	<0.001

## 6 Discussion

This study investigated the prognosis and risk factors of patients with stage I GC who had undergone radical gastrectomy. Consistent with previous studies [13], the results showed that stage I GC had an excellent prognosis with a 5-year DFS rate of over 90%. However, the prognosis was varied among different subgroups. IA (T1N0) had the best prognosis, followed by T2N0 and T1N1. After an analysis of the 12 most common clinicopathological factors, the pT and pN stages were considered to be independent risk factors for stage I GC prognosis. It is worth noting that the danger coefficient of the pN stage was higher than that of the pT stage in stage I.

Park et al. [4] identified six independent risk factors influencing the prognosis of patients with stage I GC, which include age over 65 years, male, stage IB, lymphovascular invasion, perineural invasion, and an elevated carcinoembryonic antigen level. However, Zhao [14] reported that only the pN stage could independently predict the prognosis of early GC patients. A study by In et al. [15] found that higher tumor grade, tumor located in the cardia, and inadequate lymph node dissection (<15 lymph nodes) were associated with poor overall survival. Furthermore, a study by Araki showed that venous invasion was the only independent prognostic factor for survival of patients with T2N0 [16]. An extranodal extension was also considered as a risk factor for stage IB patients [12]. Consistent with our results, T2N0 GC had a better survival rate than T1N1 GC. Wang and his colleagues analyzed nearly 2,000 stage IB GC patients who had underwent radical surgery in a Surveillance, Epidemiology and End Results (SEER) database [5]. Additionally, they demonstrated that when lymph nodes were sufficiently dissected (>15 lymph nodes), both T1N1 and T2N0 had similar survival.

The efficacy of chemotherapy in the treatment of early GC has always been controversial. Due to the excellent prognosis of early GC, most clinical trials investigating the efficacy of postoperative chemotherapy for GC excluded patients with stage I. While this study also could not confirm chemotherapy as an independent prognostic factor for stage I GC, patients with chemotherapy showed a better 5-year DFS rate compared with the patients without chemotherapy in T1N1, but the difference was not statistically significant. We inferred that the slight increase in the DFS rate is related to the excellent prognosis of patients with stage I but longer follow-up and larger study population might be needed. Some previous studies had shown that chemotherapy can potentially improve the prognosis of high-risk patients with stage I GC, even when including lymph nodes metastasis, inadequate lymph node dissection, and extranodal extension [5,12,17].

As lymph node metastasis and local invasion can reduce the DFS rate, we further investigated the clinicopathologic features associated with lymph node metastasis or depth of invasion. In this study, it was interesting to find that the younger patients were more likely to have lymph node metastasis, while older patients are more likely to have a deeper tumor invasion. While the mechanism of the phenomenon was still unclear, Zheng also reported that patients younger than 50 years of age had a higher possibility of lymph node metastasis than older patients [18]. Contrary to those studies, a recent meta-analysis showed that older patients (>60 years) are more likely to have lymph node metastasis instead, but this meta-analysis consisted of only four eligible studies and considering the selection bias as well as the publication bias, the evidence was insufficiently robust [19]. Consistent with previous studies, there was no doubt that tumor size was an independent risk factor for lymph node metastasis and deeper depth tumor invasion [19,20]. However, our study showed that tumor size was not associated with the prognosis of patients with stage I GC. Therefore, we concluded that tumor size did not affect the prognosis directly but rather indirectly through other mechanisms, such as lymph node metastasis or depth tumor invasion [5,14]. To date, the TNM staging system does not adopt tumor size as a staging indicator.

This study reported an advantage for males with respect to lymph node metastasis, which was observed more often in females. Reviewing previous studies, gender differences were noted in varying degrees of lymph node metastasis, but in most multivariate analyses, most published results showed no statistical significance. Interestingly, Zhao et al. pooled 16 studies and came to a conclusion consistent with ours [19]. We hypothesized that sex hormones might play an important role in lymph node metastasis, but the pathogenesis between gender and lymph node metastasis remained unknown. Lymphovascular invasion is a recognized risk factor for lymph node metastasis, which was confirmed again in this study, but it was not associated with patients’ prognosis. Although several studies showed that lymphovascular invasion was a risk factor for the prognosis of stage I GC, a greater number of studies concluded that lymphovascular invasion is not directly related to patients’ prognosis [4,14,16,21].

We also found that tumor location and perineural invasion were correlated with the depth of tumor invasion for stage I GC. A study showed that the overall survival of young patients with tumors located in the upper or middle third was significantly lower than those with tumor located in the lower third [22]. Several scholars believed that upper-third GC patients experienced a more aggressive disease course and suffered a worse prognosis, likely due to the pathological predominance of poorly differentiated or undifferentiated cells, which were more frequently observed in the upper-third GC patients [23,24,25]. A large meta-analysis showed that primary tumors located in the upper third of the stomach are likely to be a poor risk factor for DFS [26]. Deng et al. [27] and Aurello et al. [28] reported that perineural invasion was an independent prognostic factor for GC patients and its effect was independent of lymph node status, tumor size, and the depth of invasion as well as a range of other biological variables in the multivariate analysis. In this study, perineural invasion was associated with the depth of invasion, but the former was not directly related to the prognosis. This study reviewed previous relevant studies and summarized the potential risk factors for stage I GC (early GC), as shown in Supplementary Table S2.

There were some limitations in the present study due to its retrospective nature, but despite such limitations, we took efforts to create a clinically and scientifically sound experiment design. First, due to database limitations, the clinicopathological characteristics did not contain molecular markers, such as HER-2 status, mismatch repair deficiency, Epstein-Barr virus, and PD-1/PD-L1 expression. Furthermore, the chosen chemotherapy regimen for patients with stage I GC was not standardized. Therefore, the conclusions made in this paper require further prospective confirmation by a multicenter study with large sample size.

## 7 Conclusions

Our research demonstrates that both pT and pN stages are closely associated with the prognosis of patients with stage I GC, and the danger coefficient of the pN stage was higher than that of the pT stage. We also found that postoperative adjuvant chemotherapy might be a reasonable approach to improve the outcomes of high-risk patients, particularly in the T1N1 group.

## Abbreviations

GC: gastric cancer
DFS: disease-free survival
AJCC: American Joint Committee on Cancer
TNM: tumor-lymph node-metastasis
NCCN: National Comprehensive Cancer Network
HR: hazard ratios
95% CI: 95% confidence interval.

## References

[1] Choi IJ, Kook MC, Kim YI, Cho SJ, Lee JY, Kim CG, et al. Helicobacter pylori therapy for the prevention of metachronous gastric cancer. N Engl J Med. 2018;378(12):1085–95. 10.1056/NEJMoa1708423 29562147

[2] Ajani JA, D’Amico TA, Almhanna K, Bentrem DJ, Chao J, Das P, et al. Gastric cancer, version 3.2016, NCCN clinical practice guidelines in oncology. J Natl Compr Cancer Network: JNCCN. 2016;14(10):1286–312. 10.6004/jnccn.2016.0137 27697982

[3] He X, Wu W, Lin Z, Ding Y, Si J, Sun LM. Validation of the American Joint Committee on Cancer (AJCC) 8th edition stage system for gastric cancer patients: a population-based analysis. Gastric cancer: Off J Int Gastric Cancer Assoc Japanese Gastric Cancer Assoc. 2018;21(3):391–400. 10.1007/s10120-017-0770-1 29052053

[4] Park JH, Ryu MH, Kim HJ, Ryoo BY, Yoo C, Park I, et al. Risk factors for selection of patients at high risk of recurrence or death after complete surgical resection in stage I gastric cancer. Gastric cancer: Off J Int Gastric Cancer Assoc Japanese Gastric Cancer Assoc. 2016;19(1):226–33. 10.1007/s10120-015-0464-5 25614467

[5] Wang Y, Zhang J. Implication of lymph node staging in migration and different treatment strategies for stage T2N0M0 and T1N1M0 resected gastric cancer: a SEER population analysis. Clin Transl Oncol. 2019;21(11):1499–509. 10.1007/s12094-019-02078-y 30903518

[6] Aoyama T, Yoshikawa T, Fujikawa H, Hayashi T, Ogata T, Cho H, et al. Prognostic factors in stage IB gastric cancer. World J Gastroenterology. 2014;20(21):6580–5. 10.3748/wjg.v20.i21.6580 PMC4047344 24914380

[7] Piergiorgio F, Claudia C, Claudio B, Nunzia C, Vincenzo G. Genetic research: the role of citizens, public health and international stakeholders. Open Public Health J. 2019;12:106–13.

[8] Macdonald JS, Smalley SR, Benedetti J, Hundahl SA, Estes NC, Stemmermann GN, et al. Chemoradiotherapy after surgery compared with surgery alone for adenocarcinoma of the stomach or gastroesophageal junction. N Engl J Med. 2001;345(10):725–30. 10.1056/NEJMoa010187 11547741

[9] Mokdad AA, Yopp AC, Polanco PM, Mansour JC, Reznik SI, Heitjan DF, et al. Adjuvant chemotherapy vs postoperative observation following preoperative chemoradiotherapy and resection in gastroesophageal cancer: a propensity score-matched analysis. JAMA Oncol. 2018;4(1):31–8. 10.1001/jamaoncol.2017.2805 PMC5833647 28975352

[10] Japanese gastric cancer treatment guidelines 2014. (ver. 4). Gastric cancer: Off J Int Gastric Cancer Assoc Japanese Gastric Cancer Assoc. 2017;20(1):1–19. 10.1007/s10120-016-0622-4 PMC5215069 27342689

[11] Park JH, Kim EK, Kim YH, Kim JH, Bae YS, Lee YC, et al. Epstein–Barr virus positivity, not mismatch repair-deficiency, is a favorable risk factor for lymph node metastasis in submucosa-invasive early gastric cancer. Gastric cancer: Off J Int Gastric Cancer Assoc Japanese Gastric Cancer Assoc. 2016;19(4):1041–51. 10.1007/s10120-015-0565-1 26573601

[12] Lee IS, Kang HJ, Park YS, Ryu MH, Yook JH, Kang YK, et al. Prognostic impact of extranodal extension in stage 1B gastric carcinomas. Surgical Oncol. 2018;27(2):299–305. 10.1016/j.suronc.2018.05.014 29937185

[13] Gu L, Khadaroo PA, Chen L, Li X, Zhu H, Zhong X, et al. Comparison of long-term outcomes of endoscopic submucosal dissection and surgery for early gastric cancer: a systematic review and meta-analysis. J Gastrointest surgery: Off J Soc Surg Alimentary Tract. 2019;23(7):1493–501. 10.1007/s11605-019-04227-8 31062269

[14] Zhao BW, Chen YM, Jiang SS, Chen YB, Zhou ZW, Li YF. Lymph node metastasis, a unique independent prognostic factor in early gastric cancer. PLoS one. 2015;10(7):e0129531. 10.1371/journal.pone.0129531 PMC4496056 26154617

[15] In H, Kantor O, Sharpe SM, Baker MS, Talamonti MS, Posner MC. Adjuvant therapy improves survival for T2N0 gastric cancer patients with sub-optimal lymphadenectomy. Ann Surgical Oncol. 2016;23(6):1956–62. 10.1245/s10434-015-5075-1 26753752

[16] Araki I, Hosoda K, Yamashita K, Katada N, Sakuramoto S, Moriya H, et al. Prognostic impact of venous invasion in stage IB node-negative gastric cancer. Gastric Cancer: Off J Int Gastric Cancer Assoc Japanese Gastric Cancer Assoc. 2015;18(2):297–305. 10.1007/s10120-014-0362-2 24687437

[17] Osumi H, Yoshio T, Chin K, Ogura M, Kumekawa Y, Suenaga M, et al. Chemotherapy is effective for stage I gastric cancer in patients with synchronous esophageal cancer. Gastric Cancer: Off J Int Gastric Cancer Assoc Japanese Gastric Cancer Assoc. 2016;19(2):625–30. 10.1007/s10120-015-0517-9 26260873

[18] Zheng Z, Zhang Y, Zhang L, Li Z, Wu X, Liu Y, et al. A nomogram for predicting the likelihood of lymph node metastasis in early gastric patients. BMC cancer. 2016;16:92. 10.1186/s12885-016-2132-5 PMC4751748 26873736

[19] Zhao X, Cai A, Xi H, Chen L. Predictive factors for lymph node metastasis in undifferentiated early gastric cancer: a systematic review and meta-analysis. 2017;21(4):700–11. 10.1007/s11605-017-3364-7 28120275

[20] Hatta W, Gotoda T, Oyama T, Kawata N, Takahashi A, Yoshifuku Y, et al. A scoring system to stratify curability after endoscopic submucosal dissection for early gastric cancer: “eCura system”. Am J Gastroenterology. 2017;112(6):874–81. 10.1038/ajg.2017.95 28397873

[21] Toyokawa T, Ohira M, Sakurai K, Kubo N, Tanaka H, Muguruma K, et al. The role of adjuvant chemotherapy for patients with stage IB gastric cancer. Anticancer Res. 2015;35(7):4091–7. 26124360

[22] Liu S, Feng F, Xu G, Liu Z, Tian Y, Guo M, et al. Clinicopathological features and prognosis of gastric cancer in young patients. BMC Cancer. 2016;16:478. 10.1186/s12885-016-2489-5 PMC4946107 27418046

[23] Shim JH, Song KY, Jeon HM, Park CH, Jacks LM, Gonen M, et al. Is gastric cancer different in Korea and the United States? Impact of tumor location on prognosis. Ann Surgical Oncol. 2014;21(7):2332–9. 10.1245/s10434-014-3608-7 24599411

[24] Piso P, Werner U, Lang H, Mirena P, Klempnauer J. Proximal versus distal gastric carcinoma – what are the differences? Ann Surgical Oncol. 2000;7(7):520–5. 10.1007/s10434-000-0520-0 10947021

[25] Kunisaki C, Akiyama H, Nomura M, Matsuda G, Otsuka Y, Ono H, et al. Surgical outcomes for early gastric cancer in the upper third of the stomach. J Am Coll Surg. 2005;200(1):15–19. 10.1016/j.jamcollsurg.2004.09.015 15631915

[26] Petrelli F, Ghidini M, Barni S, Steccanella F, Sgroi G, Passalacqua R, et al. Prognostic role of primary tumor location in non-metastatic gastric cancer: a systematic review and meta-analysis of 50 studies. Ann Surgical Oncol. 2017;24(9):2655–68. 10.1245/s10434-017-5832-4 28299508

[27] Deng J, You Q, Gao Y, Yu Q, Zhao P, Zheng Y, et al. Prognostic value of perineural invasion in gastric cancer: a systematic review and meta-analysis. PLoS One. 2014;9(2):e88907. 10.1371/journal.pone.0088907 PMC3931634 24586437

[28] Aurello P, Berardi G, Tierno SM, Rampioni Vinciguerra GL, Socciarelli F, Laracca GG, et al. Influence of perineural invasion in predicting overall survival and disease-free survival in patients with locally advanced gastric cancer. Am J Surg. 2017;213(4):748–53. 10.1016/j.amjsurg.2016.05.022 27613269

